# Genetic diversity and phylogeography of broomcorn millet (*Panicum miliaceum* L.) across Eurasia

**DOI:** 10.1111/j.1365-294X.2011.05318.x

**Published:** 2011-11

**Authors:** Harriet V Hunt, Michael G Campana, Matthew C Lawes, Yong-Jin Park, Mim A Bower, Christopher J Howe, Martin K Jones

**Affiliations:** * McDonald Institute for Archaeological Research, University of CambridgeDowning Street, Cambridge CB2 3ER, UK; † Department of Biochemistry, University of CambridgeDowning Site, Tennis Court Road, Cambridge CB2 1QW, UK; ‡ Department of Plant Resources, College of Industrial Science, Kongju National UniversityYesan 340-702, Korea; § Department of Archaeology, University of CambridgeDowning Street, Cambridge CB2 3DZ, UK

**Keywords:** agricultural origins, crop phylogeography, domestication, East Asia, Eurasian steppe, microsatellites, SSRs

## Abstract

Broomcorn millet (*Panicum miliaceum* L.) is one of the world's oldest cultivated cereals, with several lines of recent evidence indicating that it was grown in northern China from at least 10 000 cal bp. Additionally, a cluster of archaeobotanical records of *P. miliaceum* dated to at least 7000 cal bp exists in eastern Europe. These two centres of early records could either represent independent domestications or cross-continental movement of this cereal that would predate that of any other crop by some 2 millennia. Here, we analysed genetic diversity among 98 landrace accessions from across Eurasia using 16 microsatellite loci, to explore phylogeographic structure in the Old World range of this historically important crop. The major genetic split in the data divided the accessions into an eastern and a western grouping with an approximate boundary in northwestern China. A substantial number of accessions belonging to the ‘western’ genetic group were also found in northeastern China. Further resolution subdivided the western and eastern genepools into 2 and 4 clusters respectively, each showing clear geographic patterning. The genetic data are consistent with both the single and multiple domestication centre hypotheses and add specific detail to what these hypotheses would entail regarding the spread of broomcorn millet. Discrepancies exist between the predictions from the genetic data and the current archaeobotanical record, highlighting priorities for investigation into early farming in Central Asia.

## Introduction

Phylogeographic studies of crop plants play an important role in understanding the population history of both the domesticated or cultivated plants themselves and the human societies responsible for their maintenance and spread. Genetic analyses of domesticates complement archaeobotanical and archaeological data in addressing major questions regarding the evolution and dispersal of cultivated plants and the (pre)history of agricultural origins and development. The crops that have received the most attention in this regard are those with the highest global economic importance today, including wheats (Giles & Brown 2006; Ozkan *et al.* 2011), barley (Morrell & Clegg 2007), rice (Vaughan *et al.* 2008) and maize (van Heerwaarden *et al.* 2011). However, there is increasing interest in the origins and diversity of so-called ‘minor crops’, many of which were staple foods across wide areas in prehistory and have inherently wide ecological tolerances (Hammer & Heller 1997; Padulosi *et al.* 1999).

In the current study, we analyse the structuring of genetic diversity in one of the world's oldest cultivated crops, broomcorn millet (*Panicum miliaceum* L.; Poaceae). *Panicum miliaceum* is a temperate representative of *Panicum*, a large and primarily pantropical genus which also includes the economically important biofuel species switchgrass (*Panicum virgatum*; McLaughlin 2005). Also known as proso, common or hog millet, *P. miliaceum* has several unique characteristics among the cereals with regard to its ecology, geography and cultivation history. It has the lowest water requirement and shortest growing season of any cereal, reaching maturity in 60–90 days after sowing (Baltensperger 2002), and a low nutrient requirement, so can be cultivated in marginal agricultural land where other cereals do not succeed. Broomcorn millet was an important cereal in many parts of Europe and Asia until recent times, and significant areas are under cultivation today both in North America, where it was introduced in the 1700s and is now grown mainly for fodder, and in the semi-arid steppe regions of Russia, northern China and Central Asia (Zohary & Hopf 2000; http://www.agroatlas.ru).

Along with foxtail millet (*Setaria italica* (L.) P. Beauv), broomcorn millet was the staple cereal of one of the world's independent centres of agricultural origins, in northern China. The earliest published record comes from the Yellow River valley site of Cishan, where diagnostic *Panicum*-type phytoliths have recently been identified in conjunction with ^14^C dates in the range 10 300–8700 cal bp (Lu *et al.* 2009). Other evidence for a significant role of broomcorn millet in the early Neolithic of north China comes from stable isotope analysis (Barton *et al.* 2009) and macrofossils (Liu *et al.* 2004) from the Loess Plateau site of Dadiwan (from 7900 cal bp) and abundant carbonized grains from the Xinglonggou site in eastern Inner Mongolia (8150–7550 cal bp; Zhao 2005a,b;). Evidence for *P. miliaceum* also occurs at a number of pre-7000 cal bp sites in eastern Europe, in the form of charred grains and grain impressions in pottery (Zohary & Hopf 2000; Hunt *et al.* 2008). This geographical distribution of early archaeobotanical findings is intriguing because of the disjunction between clusters of sites separated by several thousand km and raises questions regarding the relationship between cultivated populations in Europe and China. It has been postulated that these could represent distinct domestication episodes at either end of the Eurasian steppe region or that domestication of broomcorn millet in a single centre was followed by its spread across this wide region prior to 7000 cal bp (Jones 2004). Spread over several thousand km at this early date would be unparalleled among domesticated plants. Moreover, evidence for such spread would constitute substantially the earliest indication of contact between societies in eastern and western Eurasia. No other domesticated cereal spans this geographical range until the period from around 5000 bp, and exchange of material goods is not attested until after 4000 bp (Sherratt 2006; Jones *et al.* in review). Analysis of the phylogeography, domestic origins and spread of *P. miliaceum* are, therefore, significant not just for the crop itself but for understanding the prehistory and landscape ecology of Eurasia.

Centres of domestication of cultivated plants are often inferred from the distribution of their respective wild progenitors. The wild ancestor of broomcorn millet is not known with certainty. A weedy form, *P. miliaceum* subsp. *ruderale*, was first described from Manchuria (Kitagawa 1937) and has a widespread distribution across a region spanning from the Aralo-Caspian basin to China (Zohary & Hopf 2000; http://www.agroatlas.ru). Weedy types are also found in central Europe (Scholz 1983; Scholz & Mikoláš 1991) and in north America, where they represent a serious weed in maize crops (Bough *et al.* 1986; Colosi & Schaal 1997). These types differ from cultivated broomcorn millet by their characteristic shattering panicles (Scholz 1983) and could represent either wild ancestors or derived feral forms. Accurately identifying the nature of the evolutionary relationship between domesticated plants and related weedy forms is non-trivial (Ellstrand *et al.* 2010); currently, too little genetic data exist for *P. miliaceum* to enable resolution of this issue.

Analysis of genetic diversity in broomcorn millet is challenging because of its tetraploid genome (2n = 4× = 36) and a lack of sequence data, which has limited marker development. Previous analyses of variation in *P. miliaceum* have employed isozymes (Warwick 1987), AFLPs (Karam *et al.* 2004) and microsatellites (SSRs) transferred from other cereal species (Hu *et al.* 2009). These studies have focused on small numbers of samples and/or geographically restricted areas, and to date, no study has attempted to address large-scale phylogeographic questions in broomcorn millet.

The recent publication of 25 microsatellite loci developed *de novo* in *P. miliaceum* (Cho *et al.* 2010) has provided a more effective set of markers, which allowed us to undertake the first pan-Eurasian study of diversity and phylogeography in this crop, using landraces of *P. miliaceum* originating from across Eurasia. The use of landrace material in phylogeographic studies of cultivated plants is widely accepted as an approach that can reveal past patterns and processes (Jones *et al.* 2008b). Landraces are varieties of cultivated plants that have a historic association with a specific locality that may date back hundreds or even thousands of years. They are maintained through regeneration of seed by local farmers and are well adapted to local environmental conditions (Lister *et al.* 2010).

## Materials and methods

### Plant samples

We analysed a total of 98 accessions of *P. miliaceum* from across Eurasia. The majority of the accessions used were landraces sourced from germplasm banks (Vavilov Institute, St Petersburg, Russia; the USDA germplasm collection; and NIAS, Japan). Additional Chinese samples were collected in the field (Table 1). Pools of 8–10 individuals were bulked for the analysis of each accession. DNA was extracted from bulk seeds or seedlings using a Qiagen Plant DNeasy kit (Qiagen Ltd, Crawley, West Sussex, UK), following the manufacturer's protocols.

**Table 1:** Accessions of *Panicum miliaceum* used in this study. Location, variety and status information are as provided by the germplasm source

Accession number	Origin	Variety	Accession source and code	Status	Latitude	Longitude
MIL-1	China Beijing		USDA-ARS, PI408805			
MIL-2	Kazakhstan		USDA-ARS, PI346938			
MIL-3	Korea south		USDA-ARS, Ames 12701			
MIL-4	Kyrgyzstan		USDA-ARS, PI346936			
MIL-5	Morocco		USDA-ARS, PI517016			
MIL-6	Turkey		USDA-ARS, PI171727			
MIL-8	Georgia T'kibuli		National Museum of Georgia			
MIL-9	China NE	Atrocastaneum	VIR, #1169	Landrace		
MIL-10	China Heilongjiang	Aureum	VIR, #8804	Landrace		
MIL-11	China Shanxi	Aureum	VIR, #8964	Landrace		
MIL-12	China Shanxi	Sanguineum	VIR, #8966	Landrace		
MIL-13	China Tibet		VIR, #8973	Landrace		
MIL-14	China Lanzhou	Fulvastrum	VIR, #9100	Landrace		
MIL-15	China Heilongjiang	Griseum	VIR, #9209	Landrace		
MIL-16	Mongolia	Mongolicum, tephrum	VIR, #464	Landrace		
MIL-17	Mongolia	Tephrum	VIR, #9163	Landrace		
MIL-18	Mongolia	Tephrum	VIR, #9166	Landrace		
MIL-19	India Srinagar, Jammu and Kashmir	Flavum	VIR, #9139	Landrace		
MIL-20	India Bihar	Subcinereum	VIR, #9214	Landrace		
MIL-21	Pakistan Karachi, Sind	Flavum	VIR, #2121	Landrace		
MIL-22	Uzbekistan	Aureum	VIR, #65	Landrace		
MIL-23	Uzbekistan Bukhara	Album	VIR, #791	Landrace		
MIL-24	Kirgizstan	Flavum	VIR, #8525	Landrace		
MIL-25	Tadjikistan Gorno-Badakhshan	Flavum	VIR, #192	Landrace		
MIL-26	Turkmenistan Chardzhou	Aureum	VIR, #9055	Landrace		
MIL-27	Kazakhstan Almaty	Victoria	VIR, #2256	Landrace		
MIL-28	Kazakhstan Oskemen	Tephrum	VIR, #9559	Landrace		
MIL-29	Kazakhstan Aqtobe	Dacicum	VIR, #3773	Landrace		
MIL-30	Ukraine Kiev	Flavum	VIR, #4581	Landrace		
MIL-31	Ukraine Poltava	Flavum	VIR, #3009	Landrace		
MIL-32	Ukraine Kharkiv	Aureum	VIR, #6536	Landrace		
MIL-33	Ukraine Sumy	Aureum	VIR, #5004	Landrace		
MIL-34	Ukraine L'vov	Aureum	VIR, #9237	Landrace		
MIL-35	Ukraine Chernovtsy	Flavum	VIR, #9349	Landrace		
MIL-36	Ukraine Zakarpats'ka		VIR, #8750	Landrace		
MIL-37	Georgia	Cinereum	VIR, #2142	Landrace		
MIL-38	Azerbaijan	Coccineum	VIR, #1546	Landrace		
MIL-39	Armenia	Flavum	VIR, #1659	Landrace		
MIL-40	Russia Krasnodar	Atrocastaneum	VIR, #1500	Landrace		
MIL-41	Russia Stavropol’	Sanguineum	VIR, #1519	Landrace		
MIL-42	Russia Dagestan	Aureum	VIR, #1854	Landrace		
MIL-43	Russia Kabardino-Balkaria	Sanguineum	VIR, #1539	Landrace		
MIL-44	Russia Voronezh	Subcoccineum	VIR, #3516	Landrace		
MIL-45	Russia Kursk	Flavum	VIR, #5442	Landrace		
MIL-46	Russia Oryol	Flavum	VIR, #1733	Landrace		
MIL-47	Russia Samara		VIR, #9052	Landrace		
MIL-48	Russia Tatarstan	Flavum	VIR, #2804	Landrace		
MIL-49	Russia Saratov	Sanguineum	VIR, #3007	Landrace		
MIL-50	Russia Volgograd	Sanguineum	VIR, #7282	Landrace		
MIL-51	Russia Astrakhan’	Album	VIR, #225	Landrace		
MIL-52	Kazakhstan Oral	Victoria	VIR, #9465	Landrace		
MIL-53	Russia Orenburg	Sanguineum	VIR, #9438	Landrace		
MIL-54	Russia Altai Territory	Flavum	VIR, #2392	Landrace		
MIL-55	Russia Omsk	Vitellinum	VIR, #2825	Landrace		
MIL-56	Russia Buryatia	Tephrum	VIR, #8222	Landrace		
MIL-57	Russia Irkutsk	Mongolicum	VIR, #316	Landrace		
MIL-58	Russia Amur	Tephrum	VIR, #8508	Landrace		
MIL-59	Russia Amur	Tephrum	VIR, #8545	Landrace		
MIL-60	Russia Primorskiy Kray	Badium	VIR, #8571	Landrace		
MIL-61	Russia Primorskiy Kray	Tephrum	VIR, #50	Landrace		
MIL-62	China Gansu, Shao Dian village		Field collection	Non-agricultural		
MIL-66	Mongolia		VIR, #509	Landrace		
MIL-67	China NE	Tephrum	VIR, #1175	Landrace		
MIL-68	China NW/Manchuria [sic]	Sibiricum	VIR, #1371	Landrace		
MIL-69	China NE	Tephrum	VIR, #1999	Landrace		
MIL-70	China NE	Subcoccineum	VIR, #2012	Landrace		
MIL-71	China NW, Kashgarsky region		VIR, #2282	Landrace		
MIL-72	China NW, Kupdzha	Flavum/aureum	VIR, #2301	Landrace		
MIL-75	China	Aureum	VIR, #3750	Landrace		
MIL-76	China NW	Ochroleucum	VIR, #3790	Landrace		
MIL-77	China NE	Album	VIR, #8803	Landrace		
MIL-78	China N	Sanguineum	VIR, #8966	Landrace		
MIL-79	China NW		VIR, #9079	Landrace		
MIL-80	China NW		VIR, #9095	Landrace		
MIL-81	China NW, Shanxi	Fulvastrum	VIR, #9100	Landrace		
MIL-82	China NE, Heilongjiang	Atrocastaneum	VIR, #9205	Landrace		
MIL-83	China NE, Heilongjiang	Album	VIR, #9210	Landrace		
MIL-84	China		VIR, #10155	Landrace		
MIL-85	China		VIR, #10238	Landrace		
MIL-86	China		VIR, #10290	Landrace		
MIL-93	China Inner Mongolia,Chifeng		Field collection		41.3939°N	118.7247°E
MIL-101	China Inner Mongolia,Chifeng		Field collection		42.5359°N	120.1258°E
MIL-105	China Inner Mongolia,Chifeng		Field collection		42.0574°N	118.8090°E
MIL-106	China Inner Mongolia,Chifeng		Field collection		42.0730°N	118.8145°E
MIL-111	China Gansu, Lanzhou		Field collection		35.9479°N	103.9180°E
MIL-130	China Xinjiang, Urumqi		Field collection		43.2612°N	87.6270°E
MIL-140	Japan Hokkaido	Hankokumochi	NIAS, Japan, #3983			
MIL-143	Japan Iwate	Kokimi	NIAS, Japan, #110364	Landrace		
MIL-146	Japan Niigata	Awa	NIAS, Japan, #104909	Landrace		
MIL-148	Japan Fukushima	Mochikimi	NIAS, Japan, #74317	Landrace		
MIL-157	Japan Kochi	Kokibi	NIAS, Japan, #105018	Landrace		
MIL-160	Japan Shimane	Kogimi	NIAS, Japan, #107002	Landrace		
MIL-165	Japan Nagano	Urukibi	NIAS, Japan, #74322	Landrace		
MIL-172	Korean peninsula	Koukaizairai	NIAS, Japan, #4010	Landrace		
MIL-173	Korean peninsula	Eidougun Joumuramensan	NIAS, Japan, #4012			
MIL-174	Korean peninsula	Chuushuugunsan	NIAS, Japan, #4017			
MIL-175	Nepal	Col/Nepal/1984/32	NIAS, Japan, #54702			
MIL-176	Nepal		NIAS, Japan, #54711			

### PCR and genotyping

We undertook exploratory analyses to determine how to score the *P. miliaceum* SSR markers used and to determine polymorphic loci. These analyses used a single individual from each of 24 accessions with a wide geographic spread, constituting a subset of those in the main study. We trialled all of the 25 markers developed by Cho *et al.* (2010). Details of primer sequences are given in Table S1 (Supporting information). The forward primer of each pair was tailed with an M13 sequence to enable incorporation of a dye label following the method described by Boutin-Ganache *et al.* (2001). PCRs were carried out in 10 μL volumes containing: 1× Expand High-Fidelity PCR buffer (Roche) containing 1.5 mm MgCl_2_, 200 μm each dNTP, 0.1 μm forward primer, 0.4 μm reverse primer, 0.4 μm M13 primer labelled at the 5′ end with either FAM, PET or VIC and 0.5 U Expand High-Fidelity Polymerase (Roche). Thermal cycling conditions were as follows: 94 °C for 3 min, 30 cycles of 94 °C for 30 s, 60 °C for 45 s and 72 °C for 1 min, 10 cycles of 94 °C for 30 s, 53 °C for 45 s and 72 °C for 1 min and a final extension step of 72 °C for 10 min. These conditions were used for all loci except PaM023, which used an annealing temperature of 56 °C in the first 30 cycles. During the exploratory trials, variations in annealing temperature and MgCl_2_ concentration relative to the above protocol were also tested. PCR products were checked by electrophoresis on 1% agarose tris-borate-EDTA (TBE) gels and diluted according to concentration between 6- and 20-fold prior to analysis on an ABI3730 instrument (Applied Biosystems). Genotyping data were analysed using GeneMapper version 4.0 software (Applied Biosystems). Bins were defined for each locus, and genotypes were scored manually. Following evaluation of loci for amplification, reliability of scoring and polymorphism (see Results), the main sample set was analysed using 16 loci amplified using 15 primer pairs (Table S1, Supporting information). PCR products across these 16 loci were combined into three postplexing panels for capillary electrophoresis.

Accessions were scored at each locus for a single allele, corresponding to the largest peak, because within-accession variation is expected to be low in annual self-pollinated species (Duminil *et al.* 2009). This assumption was shown to be justified by initial analyses of 20 individuals from each of a small number of accessions (data not shown).

### Data analysis

Data were treated as haploid in downstream analyses. This approach has the advantage that analyses using diploid data usually assume Hardy–Weinberg equilibrium, which is highly likely to be violated in a strongly self-pollinated species.

The number of homogeneous genepools (*K*) was modelled using the Bayesian clustering approach implemented in the software structure version 2.3.1, using the admixture model with correlated allele frequencies, with 200 000 burnin and 1 000 000 Markov chain Monte Carlo (MCMC) reps, for 10 replicate runs of *K* = 1–10 (Pritchard *et al.* 2000; Falush *et al.* 2003, 2007). Evaluation of the optimal value of *K* followed the method described by Evanno *et al.* (2005), implemented in CorrSieve version 1.4 (Campana *et al.* 2011). Correlations of *Q* matrix output among replicate runs were checked in CorrSieve version 1.4.

We compared the output from structure by analysing the data set in instruct (Gao *et al.* 2007), which implements a similar clustering algorithm to structure but does not assume Hardy–Weinberg equilibrium and may therefore be more appropriate in cases where substantial inbreeding exists. As InStruct does not accept haploid input data, we created a false-diploid data set by duplicating each allele. As for structure, we used 200 000 burnin and 1 000 000 Markov chain Monte Carlo (MCMC) reps, for 10 replicate runs of *K =*1–10. We calculated Δ*K* (Evanno *et al.* 2005) and checked *Q* matrix correlations in CorrSieve version 1.6–5 and compared bar plots of *Q* matrix output with those from structure runs.

Diversity statistics—number of alleles, frequency of the most common allele and the measure gene diversity and polymorphic information content—were calculated in PowerMarker version 3.25 (Liu & Muse 2005). We calculated these statistics for individual loci across the whole data set and as means-across-loci for the genetic clusters identified by structure, in which samples were allocated to groups for analysis according to their highest proportional allocation to these clusters. Neighbour-joining trees, based on the genetic distance measure *D*_A_ (Nei *et al.* 1983), were constructed using the Gendist and Neighbour executables in the Phylip package (version 3.69; Joe Felsenstein, University of Washington; http://www.phylip.com). The distance measure *D*_A_ was employed as this has been shown to give the most reliable phylogenetic trees in analyses of microsatellite data for human populations (Takezaki & Nei 1996, 2008). Trees were manipulated in Dendroscope version 2.2 (Huson *et al.* 2007). Analyses of molecular variance (amovas) were performed in Arlequin 3.11 (Excoffier *et al.* 2005), grouping samples in two-level hierarchies using the clusters identified under *K =*6 and 2. amovas were carried out using the genetic distance matrix calculated by Arlequin, with 1000 permutations to test for significance of differentiation.

To determine the direction of gene flow (east to west vs. west to east), the 98 accessions were analysed using migrate 3.2.15 (Beerli 2009) under both the Maximum Likelihood and Bayesian inference paradigms (Beerli & Felsenstein 1999, 2001; Beerli 2006). The accessions were arbitrarily divided according to their geographical location into east and west populations, both containing 49 accessions, to avoid observer bias. Collected data chain lengths were allowed to vary between 50 000 and 1 000 000 iterations, with data collected every 100 steps and a burn-in equal to an additional 20% of the collected data chain length. Initial θs (mutation-scaled effective population sizes) and Ms (migration rates) were determined by estimating directly from *F*_ST_ values. Owing to point mutations in the *Panicum* microsatellite alleles, the step-wise and Brownian motion models recommended for microsatellite analyses were inappropriate. Instead, the infinite allele model was used. Relative mutation rates were estimated from the data. Maximum θs were varied between runs from 0.1 to 1000. In some Bayesian runs, the heating algorithm (both adaptive and static) was applied in order to identify stable run parameters. Other settings were left at their defaults. Convergence of chains was confirmed by rerunning analyses using the θ and M output as starting values and by inspection of chains using Tracer 1.5 (Rambaut & Drummond 2007).

## Results

The microsatellite markers developed by Cho *et al.* (2010) have not yet been widely utilized in *P. miliaceum*. For this reason, and because *P. miliaceum* is an allotetraploid species in which the relationship between the genomes is not known, and in which markers may potentially amplify two homeologous loci (Hunt *et al.* 2010), we report here the results of our evaluation of the 25 markers from Cho *et al.* (2010). One marker (PaM-085) did not amplify at all in repeated tests, and one (PaM-073) amplified inconsistently. Three markers (PaM-098, PaM-126, PaM-133) gave peak profiles that showed extensive stutter or other unexpected peaks that precluded confident interpretation and scoring, leaving a total of 20 primer pairs that yielded scorable peak profiles. Fifteen of these (PaM-004, PaM-013, PaM-014, PaM-023, PaM-025, PaM-029, PaM-031, PaM-061, PaM-096, PaM-106, PaM-107, PaM-115, PaM-117, PaM-121, PaM-134) gave peak profiles with a single set of alleles (and associated stutter peaks) indicating amplification at a single locus. Five primer pairs (PaM-060, PaM-066, PaM-094, PaM-111, PaM-145) showed two sets of peaks. These were present in all samples analysed and differed in profile shape between the two sets for each marker. We interpreted these markers as showing the fixed heterozygosity characteristic of allopolyploids (Carson 1967), with each set of alleles representing amplification in one of two homeologous loci. This is consistent with the behaviour of other nuclear markers in *P. miliaceum* (Hunt *et al.* 2010). We, therefore, designated separate ‘a’ and ‘b’ loci for each of these five markers and treated them independently. This gave a total of 25 loci with scorable profiles. Of these, nine loci (PaM-029, PaM-031, PaM-060a, PaM-060b, PaM-094b, PaM-111a, PaM-111b, PaM-117, PaM-145a) were monomorphic among the 24 samples in the exploratory analysis. These loci were, therefore, excluded from use in the main study.

The remaining 16 loci (highlighted in bold typeface in Table S1, Supporting information) were polymorphic (two or more alleles identified) in our exploratory analyses and were thus employed for the analysis of main data set. The complete data set is given in Table S2 (Supporting information). The data matrix (1568 data points) contained no null alleles or other missing data. We found a total of 78 alleles across the 16 loci. Per-locus diversity statistics are shown in Table S3 (Supporting information). The number of alleles per locus ranged from 2 to 15 (mean, 4.9). The frequency of the most common allele ranged from 0.27 to 0.98 (mean, 0.717). Mean gene diversity and polymorphic information content (PIC) were 0.391 and 0.360, respectively.

**Fig. 1:**
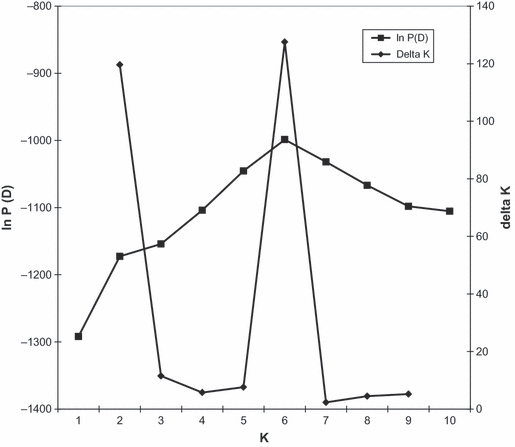
Modelling of number of genepools in *Panicum miliaceum* using structure. Ln P (D) and delta *K*, calculated according to Evanno *et al.* (2005), plotted against the number of modelled genepools (*K*).

Bayesian modelling of the number of homogeneous genepools (*K*) in structure gave a minimum mean probability of −ln P(D) = 1291.9 at *K*=1 and a maximum mean probability of −ln P(D) = 998.72 at *K*=6. Evaluation of the optimum number of *K* following the procedure by Evanno *et al.* (2005) found two clear maxima for Δ*K*, at *K*=2 and 6. The plots of ln P(D) and Δ*K* against *K* (Fig. 1), therefore, suggested that a model with two genepools captures a major split in the data, with substantial additional resolution provided under a model with *K*=6. Analysis of correspondence between replicate runs using corrsieve (Campana *et al.* 2011) showed that both these models were highly stable. Results from instruct were very similar to those from structure, with a Δ*K* maximum at *K =*2 and both *K* = 2 and 6 were stable between replicate runs (data not shown).

**Fig. 2:**
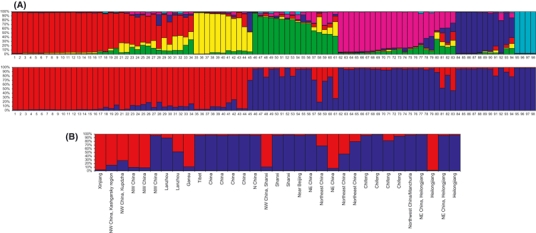
(A) Barplots of Q assignments for each sample under *K*=2 (below) and *K*=6 (top). (B) Samples from within China, on an approximate W-E gradient, showing proportional membership of each genepool under *K*=2.

Barplots of the proportional allocation in structure to each genepool for *K* = 2 and 6 are shown in Fig. 2A. The plots show that these two models relate to one another hierarchically, such that the two genepools (red and blue) in the former are subdivided into two (red and yellow) and four (blue, green, pink and cyan) clusters, respectively, in the six-genepool model. Comparison of plots using the output from instruct with those from structure showed very high consistency between the two algorithms. The main difference was that some samples allocated predominantly to the pink cluster under by structure were allocated predominantly to the green cluster by instruct and *vice versa* (data not shown). As described below, these two clusters share overlapping geographic distributions and are relatively close to one another genetically. These small differences do not, therefore, have any substantial impact on the interpretation of the results. For the following analyses, we used the output from the analyses in structure.

**Fig. 3:**
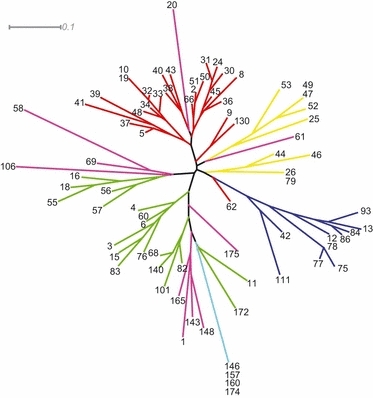
NJ tree based on Nei *et al.*’s (1983) genetic distances, excluding samples with a high level of admixture under the *K* = 6 model (highest genepool assignment value <0.6). Coloured according to the majority genepool assignment under the *K* = 6 model. Numbers refer to sampled accessions as listed in Table 1.

The neighbour-joining tree inferred from the genetic distance matrix showed moderate agreement between the relationships indicated by the phenogram topology and those from the Bayesian clustering analysis using *K =*6. This agreement improved when samples with a high degree of admixture (highest assignment proportion to any one genepool <0.6) under the six-genepool structure model are removed from the genetic distance analysis. The tree with admixed samples excluded is shown in Fig. 3.

Bootstrap resampling of the neighbour-joining tree found very low support for the internal nodes. This result is typical for genetic distance trees based on relatively few markers. To look for statistical support for phylogenetic relationships between the clusters identified by structure, we constructed a neighbour-joining tree of the genepools proposed by the *K =*6 structure model, using the modelled allele frequencies as input data with bootstrapping across loci. This tree (Fig. S1, Supporting information) indicated that the red/yellow clusters formed a clade distinct from the blue/pink/green/cyan clusters in 75% of resampled trees, consistent with the hierarchy as inferred from comparing the *K =*2 and 6 barplots. The tree also provides some support (44%) for the relationship of the pink and cyan clusters. amovas, in which the samples were grouped according to their majority proportional allocation in the structure*Q*-matrices, found significant and moderately strong genetic differentiation for both groups defined by *K =*2 (Φ_ST_ = 0.162; *P*=0.000) and by *K*=6 (Φ_ST_ = 0.324; *P*=0.000).

**Fig. 4:**
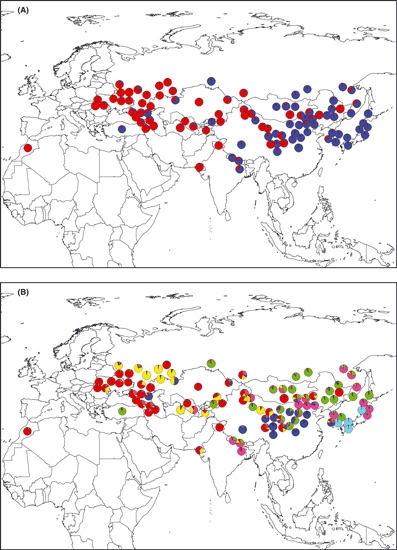
(A) Assignments of samples to two genepools under the *K* = 2 model. (B) Assignments of samples to six genepools under the *K* = 6 model. Precision of locations is highly variable: See Table 1 for details.

The genetic clusters identified by the above analyses show strong geographic structuring. The primary split in the data (*K =*2) divides the accessions into two clusters with strong respective eastern and western foci (Fig. 4A). The eastern cluster (blue) includes the majority of samples from China and Mongolia, those from Nepal and northeastern India, the Russian Far East, Korea and Japan, and a minority of five scattered samples from more westerly locations. The western cluster (red) includes the vast majority of samples from Ukraine, the Caucasus and European Russia, central Asia, northwestern India and Pakistan and ten samples from China/Mongolia. Within China, the samples appear to be geographically structured such that northeastern China is dominated by samples belonging to the blue cluster, while the majority of samples from northwestern China belong to the red cluster (Fig. 2B). However, for many Chinese accessions, the available provenance information was unspecific, and further analysis of samples with more precise location data would be needed to substantiate this pattern.

The model with six genepools, which is also strongly supported by the structure results, provides additional geographic resolution. Under this model, the blue (eastern) cluster in the two-cluster model is divided into four genepools (Fig. 2A), with the following distributions (Fig. 4B). One cluster (blue) is largely restricted to China. The second (green) appears to have its main range in northeastern China, Mongolia and Siberia but also occurs in widely scattered accessions in Turkey, Kyrgyzstan, Korea and north Japan (Hokkaido). The third genepool (pink) overlaps substantially with the green cluster both in terms of its geographical range and the topological arrangement of these samples on the neighbour-joining tree. It predominates in a number of samples from northeastern China, Nepal and northeastern India, from the Russian Far East (Amur and Primorskiy Kray) and from some samples in Japan (Honshu) and Korea. Three samples from northwestern China/Kyrgyzstan also display admixture from this genepool. The fourth cluster (cyan) is restricted to accessions from central-southern Japan and Korea. The neighbour-joining tree (Fig. 3) suggests that this genepool evolved from a split from the pink or green cluster. In the west, the red cluster under the *K =*2 model subdivides into two under *K*=6. One of these subdivisions (yellow cluster) forms a geographically coherent group of samples from northwest Kazakhstan and the Russian Plain and is also scattered in Central Asia and northwestern China. The other (red cluster) predominates in Ukraine and the Caucasus and is scattered through Morocco, Central Asia, western South Asia, China and Mongolia. With the exception of genepool 4 (cyan), the samples from China include at least one accession with a very high assignment proportion to each of the genepools, a mix that is not seen in any other region of the map.

Diversity statistics for groups of samples with the highest proportional allocation to each of the clusters identified by structure for the *K*=2 and 6 models are shown in Table 2. The results for the *K*=6 grouping show similar levels of diversity in the red, yellow, green and blue clusters. The pink cluster shows somewhat higher diversity. The cyan cluster shows no diversity, with all loci monomorphic. A comparison of the red and blue clusters defined by the *K =*2 model found somewhat higher diversity samples allocated to the blue (eastern) than to the red (western) cluster.

**Table 2:** Diversity statistics by structure cluster. A. Samples grouped by majority allocation in the *K =*6 model. B. Samples grouped by majority allocation in the *K*=2 model. Diversity statistic estimates are given correct to 2 d.p

	Number of samples with highest proportional allocation to that cluster	Diversity measures (means across 16 loci)			
		
Cluster		Number of alleles	Frequency of most common allele	Gene diversity	PIC
A. structure*K* = 6					
Blue	11	2.13	0.84	0.24	0.21
Cyan	4	1.00	1.00	0.00	0.00
Green	22	2.25	0.78	0.28	0.24
Pink	16	3.31	0.68	0.40	0.36
Red	34	2.31	0.82	0.24	0.21
Yellow	11	2.06	0.78	0.28	0.25
B. structure*K* = 2					
Blue	51	4.31	0.69	0.41	0.37
Red	47	2.88	0.79	0.29	0.25

PIC, polymorphic information content.

The results from analyses using migrate were inconclusive. Despite large final ESSes (all ≫ 1000), values for all parameters varied greatly between runs without any obvious pattern. Estimated thetas and Ms for individual runs varied both for each marker and for the final average. Repeat analyses using the same starting parameters yielded dissimilar results. This is probably because of the small number of alleles per marker (median = 4) preventing the resolution of genealogies and directions of migration, a known issue with migrate and similar programs (Beerli 2006).

## Discussion

This study constitutes the first pan-Eurasian analysis of genetic diversity in broomcorn millet. Our data show strong cross-continental phylogeographic structuring of diversity in landraces of this historically and ecologically important cereal, based on the novel and species-specific marker set developed by Cho *et al.* (2010). The primary genetic split in our data divides the 98 landrace accessions into two groups, with a similar number of accessions in each group. Moderate genetic differentiation between these two groups is supported by the amova results. The eastern (blue) group has a centre of distribution in China (Fig. 4A). These accessions likely represent the descendents of broomcorn millet originally domesticated in the same region, given that a northern Chinese centre of domestication for broomcorn millet is uncontroversial, and consistent with the archaeobotanical evidence for abundant cultivation of this crop as early as 10 000 cal bp (Liu *et al.* 2004; Zhao 2005a,b; Barton *et al.* 2009; Lu *et al.* 2009). It is uncertain whether these early findings represent forms that had already evolved domestication traits. Two types of trait are typically discernible in the archaeobotanical record for cereals, relating to spikelet morphology and grain characteristics (Jones & Brown 2007). There is some incidental reference to spikelet fragments in *P. miliaceum* in the European (but not as yet the Chinese) Neolithic. For example, Yanushevich (1989) notes that common millet at Linear Bandkeramik sites was found in ‘large quantities, preserved not only as a grain but also as chaff winnowed free of the grain and used as temper’. Such records are currently insufficient to permit comment on spikelet domestication traits. Changes in *Panicum* grain size during and beyond the Neolithic period have been observed (Zhao 2005a; Lee *et al.* 2007), and these observed variations may result from selection. While the wild ancestor is unknown, however, we lack a baseline against which to assess the expression of domestication traits.

The precise location of a centre of millet domestication within China is the subject of ongoing debate. Reports of both broomcorn and foxtail millet from Cishan and Peiligang have led to an emphasis on the central Yellow River valley as the core area of north Chinese agricultural origins. However, the presence of other early millet sites at some distance from the Yellow River valley—Dadiwan in the Loess Plateau and Xinle and Xinglonggou in the northeast—has led to suggestions that the focus of broomcorn millet domestication may have been elsewhere or that there may have been multiple foci within China (Shelach 2000; Crawford 2006; Liu *et al.* 2009). Hu *et al.* (2009) inferred a centre of broomcorn millet domestication in the Loess Plateau on the basis of a comparison of landrace genetic diversity between regions, based on SSR markers transferred from other cereal species. However, the elevated diversity among Loess Plateau landraces was not strikingly higher than that from other regions of China, its statistical significance was not evaluated and very few accessions from outside China were included in this study, so this result is equivocal. In our sample set, insufficiently precise geographical information was available for many of the accessions from China to enable us to analyse phylogeography or genetic diversity at this scale.

Our data set shows genetic diversity among Chinese landraces. Accessions with a strong proportional assignment to three of the four clusters from the *K =*6 model (pink, green and blue) are present within China, and the amova results indicate moderately strong genetic differentiation between these clusters. As shown in Table 2, there is also genetic diversity within each of these three clusters, which is at its highest in the pink cluster. From these considerations, the geographical distribution of the green and pink clusters and the archaeobotanical evidence, we infer that these groups were part of the genepool domesticated in China and spread outward in various episodes. One route of spread seems to have been northward, to Mongolia and eastern Siberia (green cluster), while the pink cluster has spread both southward, across the Himalaya, and eastward to Japan. Two distinct routes have been proposed for the introduction of cultivated plants into the Japanese archipelago: a southwesterly route via the Korean peninsula and a northeastern route into Hokkaido (Crawford & Yoshizaki 1987; Crawford & Takamiya 1990). Our data are consistent with the arrival of broomcorn millet via either or both of these routes. The phylogenetic tree indicates that the fourth subcluster within the eastern group (cyan) evolved *in situ* from the pink cluster within Japan or possibly Korea. This separation is likely to have been relatively recent, as suggested by the lack of genetic diversity within the cyan cluster (Table 2). This is consistent with the relatively late development of agriculture in Japan, which occurred from *c.* 3000 cal bp (Crawford 2011). There is some indication of spatial separation of the ancestral pink and derived cyan groups in northeastern and southwestern Japan, respectively, but further sampling is required to test this pattern.

We now consider the western (red) genetic cluster identified by the *K =*2 model. Two principal models could account for the observed distribution of this cluster across eastern Europe and Central Asia and northern China. First, this cluster could represent an independent domestication of *P. miliaceum* in eastern Europe or Central Asia. Alternatively, the red cluster may have also originated from a domestication within China and then spread westward with pioneering expansion of farming societies across the Eurasian steppe.

Considering first the model of multiple domestications, the current archaeobotanical evidence is hard to reconcile chronologically with domestication in Central Asia. The earliest record of broomcorn millet in Central Asia is from Belash in southeast Kazakhstan, where charred grains have been directly dated to 4410–4100 cal bp (Frachetti *et al.* 2010). Broomcorn millet has also been recorded from the 4th millennium cal bp site of Tahirbaj Tepe in Turkmenistan (Herrmann & Kurbansakhatov 1994), and further east, in Xinjiang, broomcorn millet grains have been recovered from Bronze Age cemeteries including Xiaohe, dated to 3600–3400 cal bp (CRAIXAR 2007). It was not among the domesticated cereals found at Jeitun in Turkmenistan (*c.* 8000 cal bp), the most extensively investigated site of the Central Asian Neolithic (Harris & Gosden 1996). However, a few Neolithic sites have been identified or excavated to date in the region.

Chronological considerations make eastern Europe a more likely candidate region than Central Asia for a second, non-Chinese, centre of domestication. However, stronger multi-faceted evidence, comparable to analyses that have recently been undertaken for Chinese sites, is needed before we can be confident that broomcorn millet was indeed cultivated in the early Neolithic of eastern Europe. Many early records in this region consist of one or a few grains (typically in assemblages including other more abundant cereals, usually wheat and barley). As noted by Crawford *et al.* (1976), ‘Great caution must be exercised in attempting to demonstrate plant husbandry at any site by the use of a single seed of a cultigen’. Although little biomolecular analysis has so far been performed, the available data from early Neolithic Ukraine do not show an isotopic signature that indicates substantial millet consumption (Lillie & Richards 2000).

Considering the genetic data, the diversity statistics indicate that the red cluster supports less diversity than the blue cluster overall. This argues against the red heartland in the west representing a centre of origin, although it should be noted that the individual subclusters (red and yellow) have similar diversity to the subclusters in the blue (eastern) group. We also note that a substantial minority (*c.* 20%) of accessions belonging to the red genepool are in China and Mongolia. It is possible that these represent an introduction from a western domestication of the red cluster, perhaps comigrating with other cereals introduced from the west in the 4th/5th millennia bp (wheats and barley; Li & Mo 2004; CRAIXAR 2007; Li *et al.* 2007). However, the penetration of western broomcorn millet into northeastern China would have required its establishment among presumably abundant and well-adapted local populations: evidence from macrofossils, stable isotopes, phytoliths and lipid biomarkers indicates that broomcorn millet was not just present, but its cultivation in China was well established at an early date (Liu *et al.* 2004; Zhao 2005a,b; Crawford *et al.* 2006; Weber & Fuller 2008; Barton *et al.* 2009; Lu *et al.* 2009; Bettinger *et al.* 2010). A third argument against a western domestication centre is the observation that the red genepool divides into two subclusters (red and yellow) under the *K*=6 model whose distribution at the westernmost end of the sample range shows a clear north/south geographic pattern (Fig. 4B). The provenance information for the samples from this region is sufficiently specific [to province or Russian *oblast* (administrative region)] that we can be confident that this is a real pattern. Genetic diversity typically exhibits strong geographic stratification at a distance from a centre of domestication, with samples from the centre of origin itself being heterogeneous for the diversity within the crop. This is consistent with the observed distribution.

We now consider the alternative major model, namely a single domestication centre in China that gave rise to all populations. The increasing dominance of the red cluster west from northern China could represent a founder effect from diverse populations in the centre of origin, paralleling the similar founder effects inferred that have led to the current distribution of the green and pink clusters (Fig. 4B). It is notable that, from the available information, it appears that the red cluster in the two-genepool model is at higher frequency in northwestern China, and the blue cluster predominates in the northeast (Fig. 2B), although more precise geographical information for Chinese accessions would be needed to explore this pattern further. The far northwest of China (Xinjiang province) has many cultural, genetic and linguistic affiliations that link it more closely to Central Asia than to the rest of modern-day China. The dominance of the red genepool in this region could represent the initial stages of a trajectory of broomcorn millet expansion west into Central Asia. Under this model, the red and yellow subclusters are not spatially separated into the eastern stages of the expansion, in the Central Asian republics, but their onward trajectory into western Russia/eastern Europe could be explained as two distinct episodes of expansion each with its own founder effect, respectively, to the southern (Caucasus and Ukraine) and northern (western Russia and Kazakhstan) parts of this territory. This is supported by the amova results, showing that these two subclusters are genetically well-differentiated.

The hypothesis of a single centre of origin for broomcorn millet in China, followed by its westward expansion, would imply that this crop reached Central Asia prior to its arrival in eastern Europe. As discussed above, this entails chronological inconsistencies with the published archaeobotanical data. This emphasizes the need for increased research both on the chronology and nature of the Neolithic in Central Asia and on evidence for cultivation and use of broomcorn millet in early Neolithic Europe. It is undisputed that broomcorn millet became a significant cereal in many parts of Europe from the Bronze Age onward (Zohary & Hopf 2000), but the role of minor crops in the early Neolithic, alongside the major staples from the Fertile Crescent, wheat and barley, requires further interrogation.

The archaeobotanical and genetic data thus currently present a set of signals that are not wholly consistent with either a single or multiple domestication centres for *P. miliaceum*. Analyses to determine the direction of migration were uninformative for our data set. The genetic picture would be clarified by comparison of landrace genetic diversity with that of the wild ancestor of broomcorn millet. Analysis of microsatellite diversity in *P. miliaceum* subsp. *ruderale* could determine whether this subspecies is indeed the wild ancestor of the domesticated form, in which case the former would be expected to maintain a more diverse genepool, or a derived feral type, whose genetic diversity is a subset of domesticated *P. miliaceum*. We did not include any samples of *P. miliaceum* subsp. *ruderale* in the current study: Although morphotypes fitting the description of this taxon are reported as being widespread across the Eurasian steppe (Zohary & Hopf 2000), detailed information or samples are not easy to find. For example, *P. miliaceum* subsp. *ruderale* is not listed on the http://www.agroatlas.ru website, and no specimens are identified as belonging to this taxon in the extensive herbarium collection of *P. miliaceum* at the Royal Botanic Gardens, Kew. Appropriate field collections of weedy forms of *P. miliaceum* for genetic comparison with cultivated types are needed, but the necessary fieldwork across vast areas of Eurasia, to give a sample set from which reliable conclusions could be drawn, would require a major international collaborative project. Our demonstration of strong phylogeographic patterning in cultivated *P. miliaceum* makes fieldwork and sampling of *P. miliaceum* subsp. *ruderale* a high priority for further investigation.

Evolutionary and population processes other than the primary spread from centres of domestication may have contributed to the observed patterns of genetic variation in broomcorn millet. Firstly, population movements later than the Neolithic may have played a role, e.g. along the Silk Routes which supported trade between Europe and Asia from the 1st millennium bc (Franck & Brownstone 1986). The relative impact of particular episodes in human history or prehistory on crop phylogeography cannot easily be determined from genetic data on landrace samples. We note that the distribution of genetic clusters does not align with modern political boundaries, so the phylogeography is unlikely to reflect recent episodes of movement. Another possible factor is linkage between microsatellite loci and genes coding for adaptive traits (Nielsen *et al.* 2006). The cross-continental spread of cereals from their centres of origin may involve adaptation to novel environments and concomitant selection of genes involved in pathways such as flowering time (Jones *et al.* 2008a), and selection for culinary traits such as endosperm starch quality may also shape patterns of molecular diversity (Fan *et al.* 2008; Yu *et al.* 2011). Cultivated broomcorn millet shows considerable phenotypic variation, including variation for phenological and culinary traits (Lyssov 1975; Hunt *et al.* 2010). With the current very limited knowledge of the genome of *P. miliaceum*, relationships between these traits and genome-wide diversity in this crop remain hypotheses to be tested. Whatever the underlying mechanisms, it is clear that the spread of broomcorn millet across the Eurasian steppe region has left a marked phylogenetic signature. There is growing interest in the development of agriculture in Central Asia (Frachetti *et al.* 2010; Li *et al.* 2011) and the genetic history of broomcorn millet, putatively the first crop to traverse this region, encapsulates key questions of pathways, chronology and ecology relevant to ongoing archaeological and botanical investigation.

## Data accessibility

The full microsatellite data set is available in Table S2 (Supporting information). Sample details are provided in Table 1.

## Supporting information

**Table S1** Microsatellite loci developed by Cho et al. (2010) and tested in *Panicum miliaceum* for this study. The 16 loci indicated in bold were those used to generate the main data set.

**Table S2** Complete data set for 98 accessions at 16 microsatellite loci.

**Table S3** Microsatellite diversity by locus.

**Fig. S1** Consensus tree from 1000 bootstrap replicates showing the relationships between the genepools modelled by STRUCTURE under the *K* = 6 model, based on modelled allele frequencies.
