# Supplementary material for: Genetic diversity and phylogeography of broomcorn millet (Panicum miliaceum L.) across Eurasia
Source: Mol Ecol. 2011 Nov;20(22):4756–71. doi: 10.1111/j.1365-294X.2011.05318.x (PMC3258423; doi:10.1111/j.1365-294X.2011.05318.x)
Supplement: Supplementary file 2 [file mec0020-4756-SD2.doc]

|  | **Allele at locus** | | | | | | | | | | | | | | | |
| --- | --- | --- | --- | --- | --- | --- | --- | --- | --- | --- | --- | --- | --- | --- | --- | --- |
| **Accession** | PaM004 | PaM013 | PaM014 | PaM023 | PaM025 | PaM061 | PaM066a | PaM066b | PaM094a | PaM096 | PaM106 | PaM107 | PaM115 | PaM121 | PaM134 | PaM145b |
| **MIL-1** | 203 | 257 | 289 | 229 | 194 | 294 | 220 | 235 | 230 | 187 | 241 | 260 | 279 | 222 | 265 | 196 |
| **MIL-2** | 205 | 257 | 289 | 229 | 196 | 291 | 220 | 232 | 230 | 190 | 241 | 260 | 281 | 235 | 263 | 196 |
| **MIL-3** | 205 | 257 | 289 | 229 | 196 | 294 | 220 | 235 | 231 | 190 | 241 | 260 | 279 | 226 | 267 | 196 |
| **MIL-4** | 205 | 257 | 289 | 227 | 196 | 291 | 220 | 235 | 230 | 190 | 241 | 260 | 279 | 226 | 265 | 196 |
| **MIL-5** | 205 | 257 | 289 | 231 | 196 | 291 | 220 | 232 | 230 | 190 | 241 | 260 | 273 | 222 | 263 | 196 |
| **MIL-6** | 205 | 257 | 289 | 231 | 196 | 291 | 220 | 235 | 230 | 190 | 241 | 260 | 279 | 226 | 267 | 196 |
| **MIL-8** | 205 | 257 | 289 | 229 | 196 | 291 | 220 | 232 | 230 | 190 | 244 | 260 | 282 | 226 | 263 | 196 |
| **MIL-9** | 205 | 257 | 289 | 231 | 196 | 291 | 220 | 235 | 230 | 190 | 241 | 260 | 273 | 218 | 263 | 196 |
| **MIL-10** | 205 | 257 | 289 | 237 | 198 | 291 | 220 | 235 | 230 | 190 | 241 | 260 | 273 | 235 | 263 | 196 |
| **MIL-11** | 205 | 257 | 289 | 231 | 194 | 294 | 220 | 235 | 231 | 190 | 241 | 260 | 279 | 226 | 260 | 196 |
| **MIL-12** | 205 | 248 | 281 | 229 | 196 | 294 | 220 | 235 | 230 | 190 | 241 | 260 | 273 | 213 | 265 | 196 |
| **MIL-13** | 205 | 248 | 281 | 231 | 196 | 294 | 220 | 235 | 232 | 190 | 241 | 260 | 273 | 235 | 269 | 196 |
| **MIL-14** | 205 | 257 | 289 | 227 | 196 | 291 | 220 | 235 | 230 | 190 | 241 | 260 | 273 | 222 | 265 | 196 |
| **MIL-15** | 205 | 257 | 289 | 224 | 196 | 291 | 220 | 235 | 231 | 190 | 241 | 260 | 279 | 213 | 245 | 196 |
| **MIL-16** | 205 | 257 | 289 | 233 | 196 | 291 | 230 | 235 | 231 | 190 | 241 | 260 | 273 | 226 | 269 | 196 |
| **MIL-17** | 205 | 257 | 289 | 229 | 196 | 291 | 230 | 235 | 231 | 190 | 241 | 260 | 281 | 222 | 254 | 195 |
| **MIL-18** | 205 | 257 | 289 | 227 | 196 | 291 | 230 | 235 | 230 | 190 | 241 | 260 | 281 | 226 | 269 | 195 |
| **MIL-19** | 205 | 257 | 289 | 237 | 198 | 291 | 220 | 235 | 230 | 190 | 241 | 260 | 273 | 235 | 263 | 196 |
| **MIL-20** | 207 | 257 | 289 | 229 | 196 | 291 | 220 | 232 | 229 | 190 | 222 | 260 | 281 | 218 | 267 | 196 |
| **MIL-21** | 205 | 257 | 289 | 229 | 196 | 291 | 220 | 235 | 230 | 190 | 239 | 260 | 273 | 222 | 263 | 196 |
| **MIL-22** | 205 | 257 | 289 | 229 | 196 | 291 | 220 | 232 | 230 | 190 | 241 | 260 | 281 | 222 | 263 | 196 |
| **MIL-23** | 205 | 257 | 289 | 229 | 196 | 291 | 220 | 235 | 230 | 190 | 249 | 260 | 273 | 218 | 261 | 196 |
| **MIL-24** | 205 | 257 | 289 | 229 | 198 | 291 | 220 | 232 | 230 | 190 | 244 | 260 | 281 | 222 | 265 | 196 |
| **MIL-25** | 205 | 257 | 289 | 229 | 198 | 291 | 220 | 235 | 230 | 190 | 231 | 262 | 275 | 222 | 263 | 196 |
| **MIL-26** | 205 | 257 | 289 | 229 | 196 | 291 | 220 | 235 | 231 | 190 | 247 | 260 | 273 | 222 | 261 | 196 |
| **MIL-27** | 205 | 257 | 289 | 229 | 196 | 291 | 220 | 235 | 230 | 190 | 241 | 260 | 281 | 222 | 261 | 196 |
| **MIL-28** | 205 | 257 | 289 | 237 | 196 | 294 | 220 | 235 | 230 | 190 | 241 | 260 | 273 | 222 | 265 | 196 |
| **MIL-29** | 205 | 248 | 281 | 235 | 196 | 294 | 220 | 235 | 231 | 190 | 231 | 260 | 273 | 235 | 263 | 196 |
| **MIL-30** | 205 | 257 | 289 | 229 | 198 | 291 | 220 | 232 | 230 | 190 | 244 | 260 | 281 | 222 | 263 | 196 |
| **MIL-31** | 205 | 257 | 289 | 229 | 198 | 291 | 220 | 232 | 230 | 190 | 267 | 260 | 281 | 222 | 265 | 196 |
| **MIL-32** | 205 | 257 | 289 | 237 | 196 | 291 | 220 | 235 | 230 | 190 | 241 | 260 | 273 | 235 | 263 | 196 |
| **MIL-33** | 205 | 257 | 289 | 237 | 196 | 291 | 220 | 235 | 230 | 190 | 241 | 260 | 281 | 235 | 263 | 196 |
| **MIL-34** | 205 | 257 | 289 | 237 | 196 | 291 | 220 | 232 | 230 | 190 | 241 | 260 | 281 | 235 | 263 | 196 |
| **MIL-35** | 205 | 257 | 289 | 229 | 196 | 294 | 220 | 235 | 230 | 190 | 244 | 260 | 281 | 222 | 263 | 196 |
| **MIL-36** | 205 | 257 | 289 | 229 | 196 | 291 | 220 | 232 | 230 | 190 | 244 | 260 | 273 | 222 | 263 | 196 |
| **MIL-37** | 205 | 257 | 289 | 231 | 196 | 291 | 220 | 232 | 230 | 190 | 241 | 260 | 273 | 222 | 258 | 196 |
| **MIL-38** | 205 | 257 | 289 | 237 | 196 | 291 | 220 | 232 | 230 | 190 | 241 | 260 | 281 | 222 | 265 | 196 |
| **MIL-39** | 205 | 257 | 295 | 237 | 194 | 291 | 220 | 232 | 230 | 190 | 241 | 260 | 273 | 222 | 263 | 196 |
| **MIL-40** | 205 | 257 | 289 | 231 | 196 | 291 | 220 | 232 | 230 | 190 | 241 | 260 | 281 | 205 | 265 | 196 |
| **MIL-41** | 207 | 257 | 289 | 237 | 198 | 291 | 220 | 232 | 230 | 190 | 241 | 260 | 273 | 222 | 260 | 196 |
| **MIL-42** | 205 | 248 | 281 | 229 | 196 | 291 | 220 | 235 | 230 | 190 | 241 | 260 | 281 | 222 | 265 | 196 |
| **MIL-43** | 205 | 257 | 289 | 237 | 196 | 291 | 220 | 232 | 230 | 190 | 241 | 260 | 281 | 218 | 260 | 196 |
| **MIL-44** | 205 | 257 | 289 | 229 | 196 | 291 | 220 | 235 | 231 | 190 | 241 | 260 | 281 | 230 | 265 | 196 |
| **MIL-45** | 205 | 257 | 289 | 229 | 196 | 291 | 220 | 232 | 230 | 190 | 244 | 260 | 281 | 222 | 263 | 196 |
| **MIL-46** | 205 | 257 | 289 | 229 | 196 | 294 | 220 | 235 | 231 | 190 | 244 | 260 | 281 | 230 | 263 | 196 |
| **MIL-47** | 205 | 257 | 289 | 229 | 196 | 291 | 220 | 235 | 231 | 190 | 231 | 262 | 281 | 235 | 261 | 196 |
| **MIL-48** | 205 | 257 | 289 | 237 | 196 | 291 | 220 | 232 | 230 | 190 | 241 | 260 | 273 | 235 | 263 | 196 |
| **MIL-49** | 205 | 257 | 289 | 229 | 196 | 291 | 220 | 235 | 231 | 190 | 231 | 262 | 281 | 235 | 261 | 196 |
| **MIL-50** | 205 | 257 | 289 | 229 | 196 | 291 | 220 | 232 | 230 | 190 | 241 | 260 | 281 | 235 | 261 | 196 |
| **MIL-51** | 205 | 257 | 289 | 229 | 196 | 291 | 220 | 232 | 230 | 190 | 241 | 260 | 281 | 235 | 263 | 196 |
| **MIL-52** | 205 | 257 | 289 | 231 | 196 | 291 | 220 | 235 | 231 | 190 | 231 | 262 | 281 | 235 | 263 | 196 |
| **MIL-53** | 205 | 257 | 289 | 231 | 196 | 291 | 220 | 235 | 230 | 190 | 231 | 262 | 281 | 230 | 265 | 196 |
| **MIL-54** | 205 | 257 | 289 | 231 | 196 | 294 | 220 | 232 | 230 | 190 | 241 | 260 | 273 | 222 | 261 | 196 |
| **MIL-55** | 205 | 257 | 289 | 233 | 196 | 291 | 230 | 235 | 231 | 190 | 241 | 260 | 281 | 226 | 269 | 195 |
| **MIL-56** | 205 | 257 | 289 | 229 | 196 | 291 | 230 | 235 | 230 | 190 | 241 | 260 | 279 | 226 | 267 | 196 |
| **MIL-57** | 205 | 257 | 289 | 229 | 196 | 291 | 230 | 235 | 231 | 190 | 241 | 260 | 279 | 222 | 267 | 196 |
| **MIL-58** | 207 | 257 | 289 | 231 | 194 | 291 | 230 | 235 | 230 | 190 | 239 | 260 | 273 | 235 | 269 | 195 |
| **MIL-59** | 205 | 257 | 289 | 229 | 196 | 291 | 230 | 235 | 230 | 190 | 244 | 260 | 279 | 222 | 258 | 196 |
| **MIL-60** | 205 | 257 | 289 | 231 | 196 | 291 | 220 | 235 | 230 | 190 | 241 | 260 | 279 | 226 | 267 | 196 |
| **MIL-61** | 205 | 257 | 289 | 233 | 194 | 291 | 220 | 235 | 230 | 190 | 244 | 260 | 281 | 222 | 270 | 196 |
| **MIL-62** | 205 | 257 | 289 | 229 | 196 | 291 | 220 | 235 | 230 | 190 | 241 | 260 | 273 | 222 | 260 | 196 |
| **MIL-66** | 205 | 257 | 289 | 229 | 196 | 291 | 220 | 232 | 230 | 190 | 241 | 260 | 281 | 222 | 263 | 196 |
| **MIL-67** | 205 | 257 | 289 | 229 | 196 | 291 | 230 | 235 | 230 | 190 | 244 | 260 | 281 | 226 | 258 | 196 |
| **MIL-68** | 205 | 257 | 289 | 231 | 196 | 294 | 220 | 235 | 230 | 190 | 241 | 260 | 279 | 226 | 245 | 196 |
| **MIL-69** | 205 | 257 | 289 | 229 | 194 | 291 | 230 | 235 | 230 | 190 | 241 | 260 | 273 | 218 | 260 | 196 |
| **MIL-70** | 205 | 257 | 289 | 229 | 196 | 291 | 220 | 235 | 230 | 190 | 241 | 260 | 273 | 222 | 265 | 195 |
| **MIL-71** | 205 | 257 | 289 | 231 | 196 | 291 | 220 | 235 | 230 | 190 | 244 | 260 | 273 | 218 | 265 | 196 |
| **MIL-72** | 203 | 257 | 289 | 231 | 196 | 291 | 220 | 235 | 230 | 190 | 241 | 260 | 273 | 222 | 261 | 196 |
| **MIL-75** | 205 | 243 | 275 | 229 | 196 | 294 | 220 | 235 | 232 | 190 | 241 | 260 | 279 | 230 | 265 | 196 |
| **MIL-76** | 205 | 257 | 289 | 231 | 196 | 291 | 220 | 235 | 231 | 190 | 241 | 260 | 279 | 226 | 249 | 196 |
| **MIL-77** | 205 | 243 | 275 | 229 | 196 | 294 | 220 | 235 | 232 | 190 | 241 | 260 | 279 | 222 | 265 | 196 |
| **MIL-78** | 205 | 248 | 281 | 229 | 196 | 294 | 220 | 235 | 230 | 190 | 241 | 260 | 273 | 213 | 265 | 196 |
| **MIL-79** | 205 | 257 | 289 | 229 | 196 | 291 | 220 | 235 | 231 | 190 | 247 | 260 | 273 | 222 | 261 | 196 |
| **MIL-80** | 205 | 257 | 289 | 231 | 196 | 291 | 220 | 235 | 230 | 190 | 239 | 260 | 281 | 222 | 263 | 196 |
| **MIL-81** | 205 | 257 | 289 | 229 | 196 | 291 | 220 | 235 | 230 | 190 | 241 | 260 | 273 | 222 | 265 | 196 |
| **MIL-82** | 205 | 257 | 289 | 231 | 196 | 294 | 220 | 235 | 230 | 190 | 241 | 260 | 279 | 226 | 265 | 196 |
| **MIL-83** | 205 | 257 | 289 | 224 | 196 | 294 | 220 | 235 | 231 | 190 | 241 | 260 | 279 | 226 | 245 | 196 |
| **MIL-84** | 205 | 248 | 281 | 231 | 196 | 294 | 220 | 235 | 232 | 190 | 241 | 260 | 273 | 222 | 265 | 196 |
| **MIL-85** | 205 | 257 | 289 | 231 | 196 | 294 | 220 | 235 | 232 | 190 | 241 | 260 | 279 | 222 | 267 | 196 |
| **MIL-86** | 205 | 248 | 281 | 229 | 196 | 294 | 220 | 235 | 232 | 190 | 241 | 260 | 273 | 222 | 265 | 196 |
| **MIL-93** | 205 | 248 | 281 | 231 | 196 | 294 | 220 | 235 | 232 | 190 | 241 | 260 | 273 | 226 | 267 | 196 |
| **MIL-101** | 205 | 257 | 289 | 231 | 196 | 294 | 220 | 235 | 230 | 190 | 241 | 260 | 279 | 222 | 265 | 195 |
| **MIL-105** | 205 | 257 | 289 | 231 | 196 | 294 | 220 | 235 | 230 | 190 | 241 | 260 | 273 | 222 | 265 | 195 |
| **MIL-106** | 235 | 257 | 289 | 236 | 196 | 291 | 230 | 235 | 229 | 190 | 244 | 260 | 275 | 291 | 241 | 196 |
| **MIL-111** | 205 | 248 | 281 | 229 | 196 | 291 | 220 | 235 | 230 | 187 | 241 | 260 | 273 | 222 | 260 | 196 |
| **MIL-130** | 205 | 257 | 289 | 231 | 196 | 291 | 220 | 235 | 230 | 190 | 244 | 260 | 273 | 222 | 263 | 196 |
| **MIL-140** | 205 | 257 | 289 | 233 | 196 | 294 | 220 | 235 | 230 | 190 | 241 | 260 | 279 | 226 | 245 | 196 |
| **MIL-143** | 207 | 257 | 289 | 229 | 194 | 294 | 220 | 235 | 230 | 190 | 241 | 260 | 279 | 218 | 256 | 196 |
| **MIL-146** | 207 | 257 | 289 | 225 | 194 | 294 | 220 | 235 | 232 | 190 | 239 | 260 | 281 | 226 | 238 | 196 |
| **MIL-148** | 207 | 257 | 289 | 233 | 194 | 294 | 220 | 235 | 230 | 190 | 241 | 260 | 279 | 243 | 261 | 196 |
| **MIL-157** | 207 | 257 | 289 | 225 | 194 | 294 | 220 | 235 | 232 | 190 | 239 | 260 | 281 | 226 | 238 | 196 |
| **MIL-160** | 207 | 257 | 289 | 225 | 194 | 294 | 220 | 235 | 232 | 190 | 239 | 260 | 281 | 226 | 238 | 196 |
| **MIL-165** | 205 | 257 | 289 | 236 | 194 | 294 | 220 | 235 | 230 | 190 | 241 | 260 | 279 | 218 | 265 | 196 |
| **MIL-172** | 205 | 257 | 289 | 231 | 194 | 294 | 220 | 235 | 230 | 190 | 239 | 260 | 279 | 209 | 250 | 196 |
| **MIL-173** | 205 | 257 | 289 | 229 | 196 | 294 | 220 | 235 | 232 | 190 | 241 | 260 | 279 | 222 | 263 | 196 |
| **MIL-174** | 207 | 257 | 289 | 225 | 194 | 294 | 220 | 235 | 232 | 190 | 239 | 260 | 281 | 226 | 238 | 196 |
| **MIL-175** | 205 | 257 | 289 | 213 | 196 | 294 | 220 | 235 | 230 | 190 | 241 | 260 | 273 | 209 | 245 | 196 |
| **MIL-176** | 205 | 257 | 289 | 229 | 196 | 291 | 220 | 235 | 229 | 190 | 241 | 260 | 281 | 222 | 245 | 196 |
