# Supplementary figures and images for: Genetic diversity and phylogeography of broomcorn millet (Panicum miliaceum L.) across Eurasia
Source: Mol Ecol. 2011 Nov;20(22):4756–71. doi: 10.1111/j.1365-294X.2011.05318.x (PMC3258423; doi:10.1111/j.1365-294X.2011.05318.x)

└─ 100.0

pink

cyan

455

green

287

blue

760

yellow

red

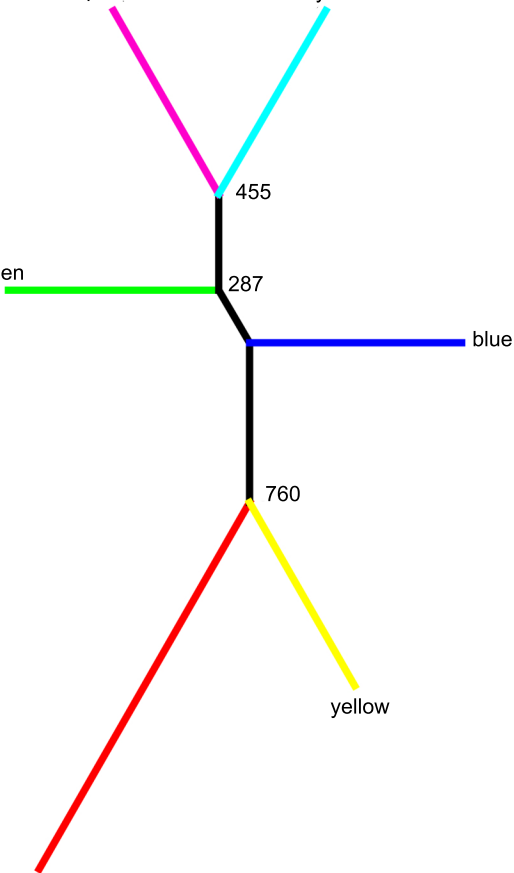

Supplement: Supplementary file 4 [file mec0020-4756-SD4.pdf]
